# Green Christmas: bryophytes as ornamentals in Croatian traditional nativity scenes

**DOI:** 10.1186/s13002-022-00516-w

**Published:** 2022-03-10

**Authors:** Marija Bučar, Vedran Šegota, Anja Rimac, Nikola Koletić, Tihana Marić, Antun Alegro

**Affiliations:** 1 Division of Botany, Department of Biology, Faculty of Science, University of Zagreb, Marulićev trg 20/II, 10000 Zagreb, Croatia; 2 Department of Pharmaceutical Botany, Faculty of Pharmacy and Biochemistry, University of Zagreb, Schrottova 39, 10000 Zagreb, Croatia

**Keywords:** Bryophytes, Christmas, Croatia, Ethnobryology, Farmers’ market, Nativity scene

## Abstract

**Background:**

The bryophytes are a plant group that is smaller than and not as well known as the vascular plants. They are less used and are almost completely neglected in ethnobotanical studies. Traditional nativity scenes depicting the birth of Christ are commonly decorated with both vascular plants and bryophytes. The aim of this study was to document the diversity of decorative bryophytes sold during the Advent season at farmers’ markets in Croatia (Southeastern Europe, Balkan Peninsula).

**Methods:**

Twenty-eight farmers’ markets in the two largest Croatian cities (Zagreb in the continental part and Split in the Mediterranean part) were studied in the search for local vendors selling bryophytes during the pre-Christmas season. The bryophytes collected were identified and analysed with respect to families, growth type, life forms and threat status.

**Results:**

Among 275 collected specimens, 43 moss and four liverwort species were identified. The mean number of species per vendor was 3.5. The most frequent species were *Hypnum cupressiforme*, *Homalothecium sericeum* and *Ctenidium molluscum*. Mats, wefts and tufts were the most common life-forms, while pleurocarpous prevailed over acrocarpous mosses, as they are usually pinnately branched and form large carpets, suitable for decorations. The overall selection of bryophytes and the decorations made of them were more diverse and abundant in inland Croatia, where 49 vendors at 15 farmers’ markets sold goods containing 43 species. In Mediterranean Croatia, at six farmers’ markets only 29 vendors sold goods, which contained 18 species. A considerable number of species that are less attractive to harvesters were collected non-intentionally, entangled in carpets of other, more frequent species. Among them, *Rhodobryum ontariense* and *Loeskeobryum brevirostre* are rare and insufficiently recorded in Croatia so far.

**Conclusions:**

The present study provided a first perspective on the use of bryophytes in traditional nativity scenes in Croatia and Southeastern Europe, contributing to scarce ethnobotanical documentation of the decorative use of bryophytes in Christmas festivities in Europe and globally.

## Background

Bryophytes are a phylogenetically old group of relatively small and inconspicuous plants that are considerably less known than the bigger, more conspicuous vascular plants and are in consequence less used and studied [1, 2]. However, some bryophytes have found their place in ethnobotany around the world. China, India, Mexico and the USA are the countries with the most ethnobryological records. In China, bryophytes were mainly a part of the Chinese traditional medicine system, Native North Americans used them for medical purposes, while in Mexico they were used for ceremonial, craft, medical and environmental purposes [2, 3]. In some cultures, mosses were used or sometimes are still used for construction of pavements, as fillers in wall cracks, fire insulation, chinking, sound insulation and similar purposes [4].

Even though it has been scientifically proven that some bryophytes show a potential for agricultural and medicinal use due to their antimicrobial, antifeedant and even antitumor properties [4, 5], most scientific research of this kind is still focused on vascular plants. The commercial value of bryophytes today seems to be limited to ornamental uses (e.g. moss gardening), peat (*Sphagnum* L. ssp.) or biofuel production, bioremediation and biomonitoring [4, 6]. In the decorative greens industry and floral trade, mosses are used for funerals, Christmas greens, flower show displays and moss green walls. Bryophytes are recognized as good bioindicators of environmental conditions such as soil and water pH, water nutrient content (aquatic mosses), acid rain and the presence of heavy metals [7–9]. Their ability to accumulate heavy metals makes some bryophyte species particularly useful in bioremediation. This is why they can be used for pollution studies, waste treatment and soil conditioning. Mosses that form mat-like thalloids help prevent soil erosion, while in forests they create seed beds influencing tree growth by either enhancing or prohibiting seedling survival [4, 10].

Records of commercial moss harvesting exist in studies from the Appalachian Mountains of West Virginia [11, 12] and Oregon [13] in the USA, and the Monarch Butterfly Biosphere Reserve, Sierra Chincua in Mexico [14]. Although bryophytes are nowadays largely used for decoration in the floral industry, their traditional decorative usage has been almost completely unrecorded. The decorative use of mosses has been only recently documented among farmers’ families in the Napf region in Switzerland [15]. The mosses are still used in a very peculiar custom from western Spain (Béjar, Province of Salamanca) which takes place during the Corpus Christy festivity and includes a procession in which a significant role is played by so-called Moss Men—men dressed in costumes made of moss carpets. This custom celebrates the victory of the Christians over the Muslims from the thirteenth century when people of Béjar used moss mats as camouflage [16].

One of the widespread uses of bryophytes is ornamental as part of the tradition of making nativity scenes (manger scenes, nativity sets) during the Christmas holidays. This custom of preparing dioramas representing the events immediately before and after the birth of Christ exists in most countries with Christian populations. It is believed that it was established in France and Italy by the Franciscan Order in the thirteenth century, subsequently spreading across the globe [17, 18]. Mosses are used to simulate vegetation in the nativity scenes by putting moss carpets on the stable roof, in baby Jesus’ cradle or around the nativity. In Spain, the use of mosses for covering the elements that represent the mountains in the Nativity scenes has been widely recorded. Here, the moss carpets are used to simulate fields, scrubland and forests, while in arid sceneries, they are usually set along the rivers, shaping the lush riverside vegetation [18]. In Mexico, the tradition of using wild gathered plants (including bryophytes) for Christmas decorations dates back to the sixteenth century, and the building of the nativity scenes in private homes is not only an act of religious devotion but also a demonstration of social prestige [17].

Nativity scenes have a long tradition in Croatia dating back to the seventeenth century. This tradition was mainly imported from Central Europe enhanced by Roman Catholic monastic orders of Franciscans and Jesuits, on the one hand, and by court festivities copied by local nobility on the other [19]. Initially, nativity scenes were mainly exhibited in churches, but by the seventeenth century, there was a house nativity scene in the Croatian island of Cres, the earliest known example. During the nineteenth century nativity scenes had become a frequent feature in both peasant and bourgeois households. More or less elaborated nativity scenes included figures and imaginary landscapes of the Holy Land or even local landscapes with familiar characteristics. To arrange these landscapes mosses were ideal material due to their size and accessibility in the winter. On the other hand, greenery has an important role in Christmas symbolism, of which mosses, with evergreen species of vascular plants, are a part [19].

The use of vascular plants in Christmas festivities, unlike that of bryophytes, was often documented, from several different aspects. In terms of food preparation, several traditions were reported in Eastern Europe: the cooking of dishes using non-wild forest products and wheat (*Triticum aestivum* L.) in Ukraine [20, 21], collecting hazelnuts (*Corylus avellana* L.) from the wild in Poland [22], and the use of barley (*Hordeum vulgare* L.) for Christmas Eve gruel and of poppy (*Papaver somniferum* L.) in the Polish-Lithuanian-Belarusian borderland [23]. Additionally, a Mediterranean Christmas tradition of frying wild thistles in batter has been recently documented in Sicily [24]. In terms of ceremonial decoration, the use of fir (*Abies alba* Mill.) and spruce (*Picea abies* (L.) H. Karst.) from the wild in Austria [25], as well as extensive private cultivation of *Casuarina* L. and pines (*Pinus* L.) in Cameroon [26] for Christmas trees were registered. Besides Christmas trees, various vascular plants are used for ornamenting the nativity sets in Spain (ferns and flowering plants) [18, 28] and Mexico (bromeliads, orchids, lichens and lycopods) [17]. The ceremonial use of mistletoe (*Viscum album* L.) during the Christmas period was documented in Poland [29] and Italy [30], while various plants (hay, garlic cloves, walnuts, poppy seeds, spruce) were used for table decorations and bouquets in Ukraine [31]. Several reports of the ritual and symbolic use of plants at Christmas time, including fire and embers, are available. In Italy, torches are made of fir (*Abies alba*) [32], while grapevine wood (*Vitis vinifera* L.) is used for fires on Christmas Eve, and then allowed to burn slowly every day until Epiphany (6^th^ January), when still burning logs are placed in the vineyards (“Christmas log”) [30]. In Ukraine, the incense of *Juniperus communis* L. is prepared for Christmas [20].

Ethnobotanical studies in Croatia are of fairly recent date and have been focused mainly on wild food plants [33–39], with only sparse documentation of their decorative usage [37]. In terms of ethnobryology, Croatia and Europe in general, are very much *terra incognita*. The present study tries to offer a first perspective on the use of bryophytes in traditional nativity scenes in Croatia (Southeastern Europe, Balkan Peninsula), contributing to scarce ethnobotanical documentation of the decorative use of bryophytes in Christmas festivities in Europe and globally. According to our previous observations, in Croatian rural and suburban communities, bryophytes are traditionally collected in nature by the nativity scene creators themselves. However, in larger cities, mosses are frequently sold in local markets. In the present study, farmers’ markets were investigated, and market stands selling bryophytes for nativity sets and other decorations were examined in December (pre-Christmas period, e.g. Advent season). Our goal was to determine the overall bryophyte diversity and to identify which species are preferred by non-scientific collectors (vendors).

## Materials and methods

**Fig. 1:**
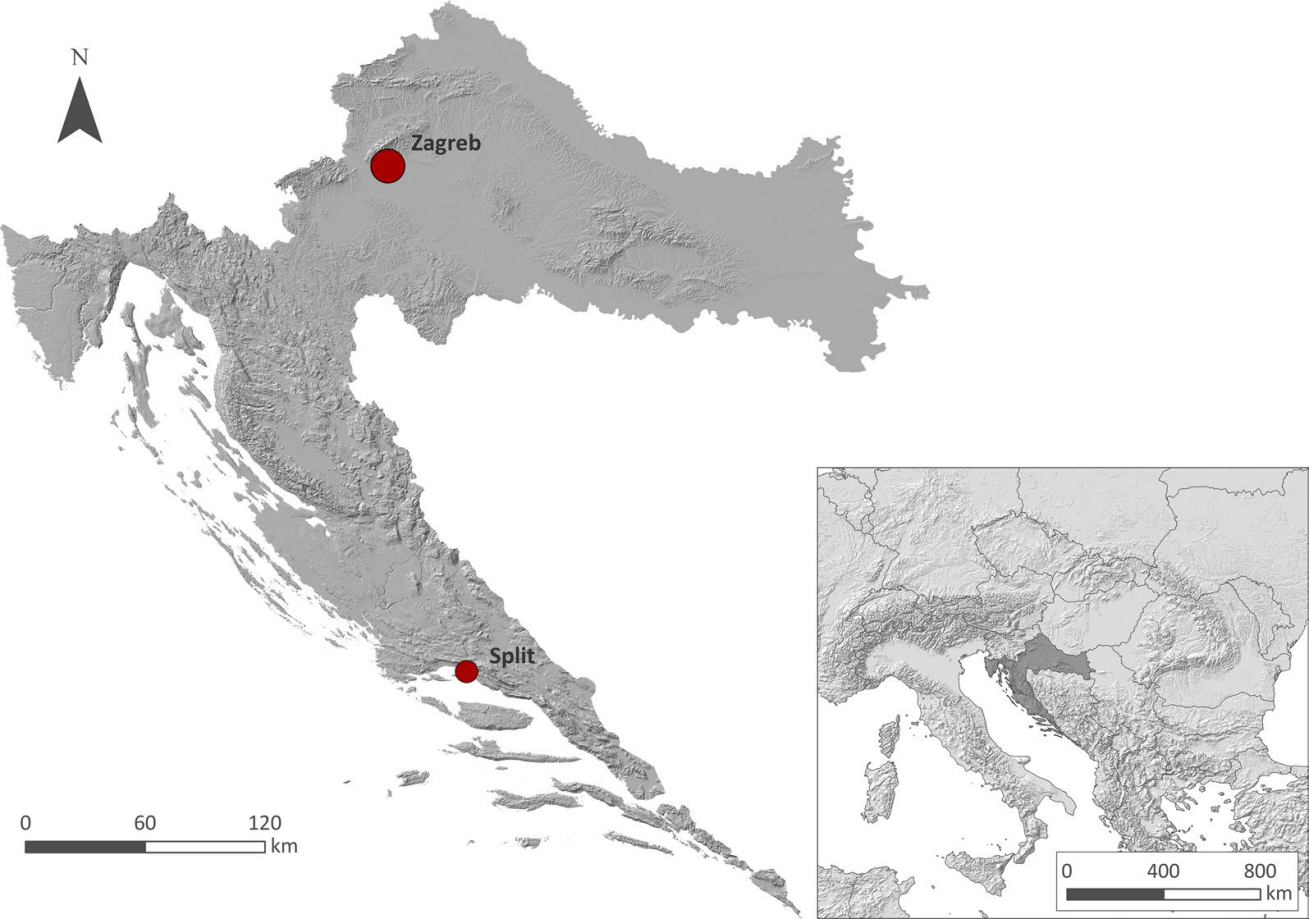
The geographic positions of the two largest Croatian cities (Zagreb and Split) in Croatia, and Croatia in Europe

**Fig. 2:**
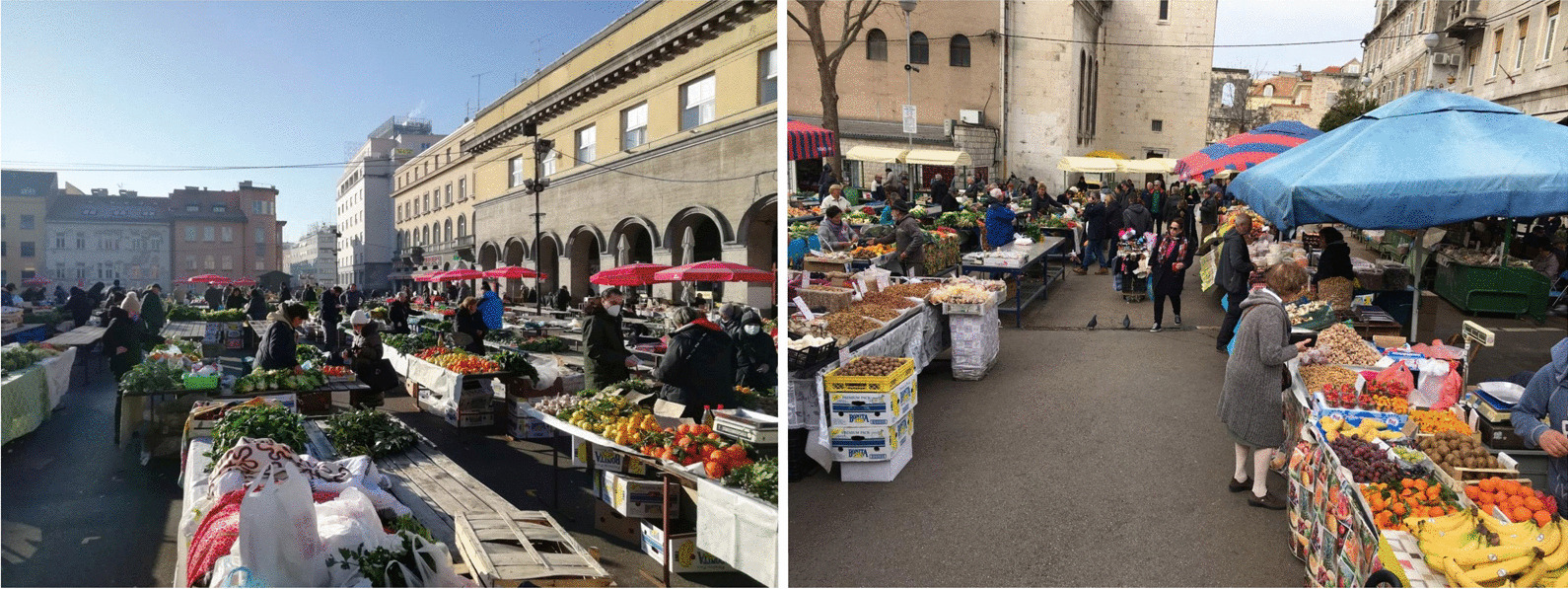
The two largest farmers’ markets in Croatia—Dolac in Zagreb (left) and Stari Pazar in Split (right). Photographs by Vedran Šegota and Katherine Pepper

The research was carried out following the Principles of Professional Responsibility of the American Anthropological Association and the International Society of Ethnobiology Code of Ethics (2006) [40, 41]. To test whether there are differences in species collecting preference between two main geographical and climatic regions of the country, the two largest Croatian cities, Zagreb (in the inland part) and Split (in the Mediterranean part), were chosen (Fig. 1, Table 1). During the Advent seasons of 2016, 2017 and 2018, a network of 28 farmers’ markets in Zagreb (22) and Split (6) was visited for sampling bryophytes offered by local vendors (Fig. 2). Data were collected using semi-structured interviews, while informants were approached during their regular sale routine. No sex or age categories were registered. It was possible to identify several bryophyte species at the markets; however, most of the specimens were sampled with owner approval or sometimes purchased, transferred to the Department of Botany of the Faculty of Science in Zagreb, where they were finally identified using identification keys [42–49]. Whenever possible, the vendors were asked about the collecting sites and habitats. Some vendors gave us only basic information and refused to take part in longer interviews, due to lack of time since they were engaged in intensive pre-Christmas selling activities. Furthermore, analysis of the species regarding the families, growth type (pleurocarpous vs. acrocarpous species), life forms and threat status was made. Nomenclature follows [50], threat status [51], and life-forms [52]. Seven categories were used: Ac—aquatic colonial, Cu—cushion, De—dendroid, Fa—fan, Ma—mat, Tu—tuft, We—weft, while life-forms of three species were not available in [51] and were assigned according to the life form of their congenital pair (*Brachythecium tommasinii*—mat, *Eurhynchium angustirete*—weft and *Rhodobryum ontariense*—tuft). Differences in species composition between the inland part of Croatia (Zagreb) and the Mediterranean part (Split) were examined.

**Table 1:** Main climatological characteristics of the areas of Zagreb for the period 1861–2020 and Split for the period 1948–2020 provided by courtesy of Croatian Meteorological and Hydrological Service and a summarized description of zonal vegetation

	Zagreb	Split
Mean annual temperature	11.6 °C	16.4 °C
Warmest month	July (21.9 °C)	July (26.1 °C)
Coldest month	January (0.6 °C)	January (7.9 °C)
Annual precipitation	886.9 mm	810.7 mm
Days with rain	128	112
Days with frost	11	1
Days with snow	23	2
Zonal vegetation	Temperate broad-leaved deciduous forests of sessile oak (*Quercus petraea* (Matt.) Liebl.), pedunculate oak (*Q. robur* L.) and beech (*Fagus sylvatica* L.)	Mediterranean evergreen holm oak (*Quercus ilex* L.) forests and scrubs

## Results and discussion

**Fig. 3:**
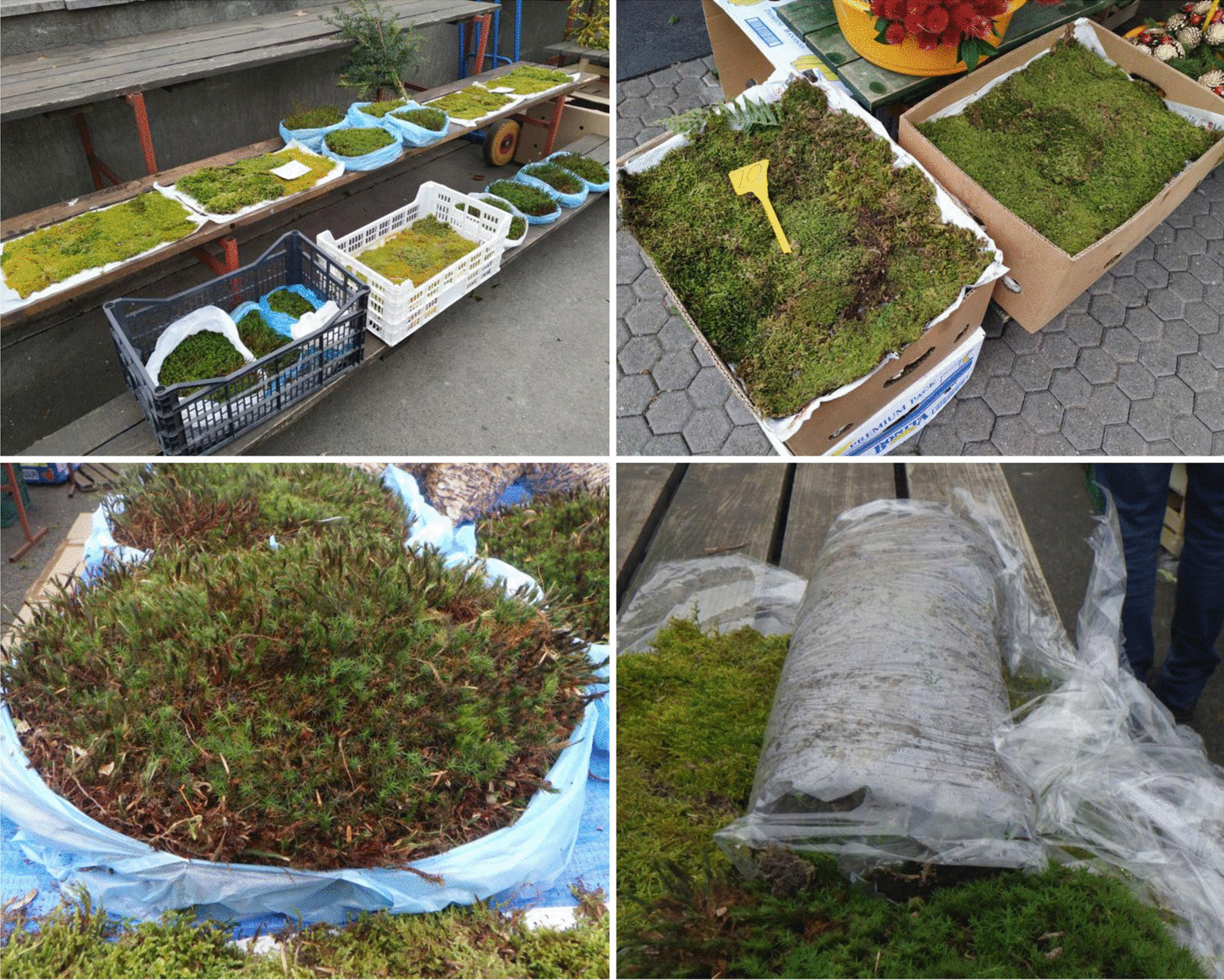
Variety of moss carpet packing (newspaper sheet-size layers in boxes, within plastic bags and rolled in plastic foil) from the market of Dolac (Zagreb). Photographs by Marija Bučar

Ninety-eight semi-structured interviews were carried out within the present study. Out of 22 markets visited in Zagreb, bryophytes were sold on 15, as seven lacked bryophytes in their offer. By contrast, all six visited markets in Split offered bryophytes. They were mostly sold in organized open-air farmer’s markets, except for two vendors in Split with more informal standalone market stalls alongside the street. Similarly, in Mexico, during December, bryophytes are regularly offered for sale in local markets all over the country [15]. In the largest Mexican Christmas plant market in the town of Oaxaca, the selling spaces are open-air and sellers place their plants in small heaps on the sidewalk in the manner of Indian market-selling [17]. Apart from Mexico, moss harvesting for commercial purposes at Christmas has also been documented in Panama [53, 54] and Spain [18], although this practice is surely widespread in other countries too. In Croatian farmers’ markets, bryophytes were most frequently offered in carpets of newspaper sheet size packed in several layers separated by newspapers and brought to markets in cardboard boxes or wooden and plastic crates (Fig. 3). This method of bundling collected newspaper-size moss carpets into packs of handling weight was reported also among moss gatherers in the state of Michoacán in Mexico [15]. In addition, in Zagreb, similar size carpets were also, although only occasionally, offered rolled in plastic foil (Fig. 3). Similarly, there were reports from North Carolina and Virginia (USA) of harvested and dried moss carpets being bundled into bales and taken to market [12]. Cushion mosses were, on the contrary, usually sold in Croatian farmers’ markets in plastic bags (Fig. 3).

**Fig. 4:**
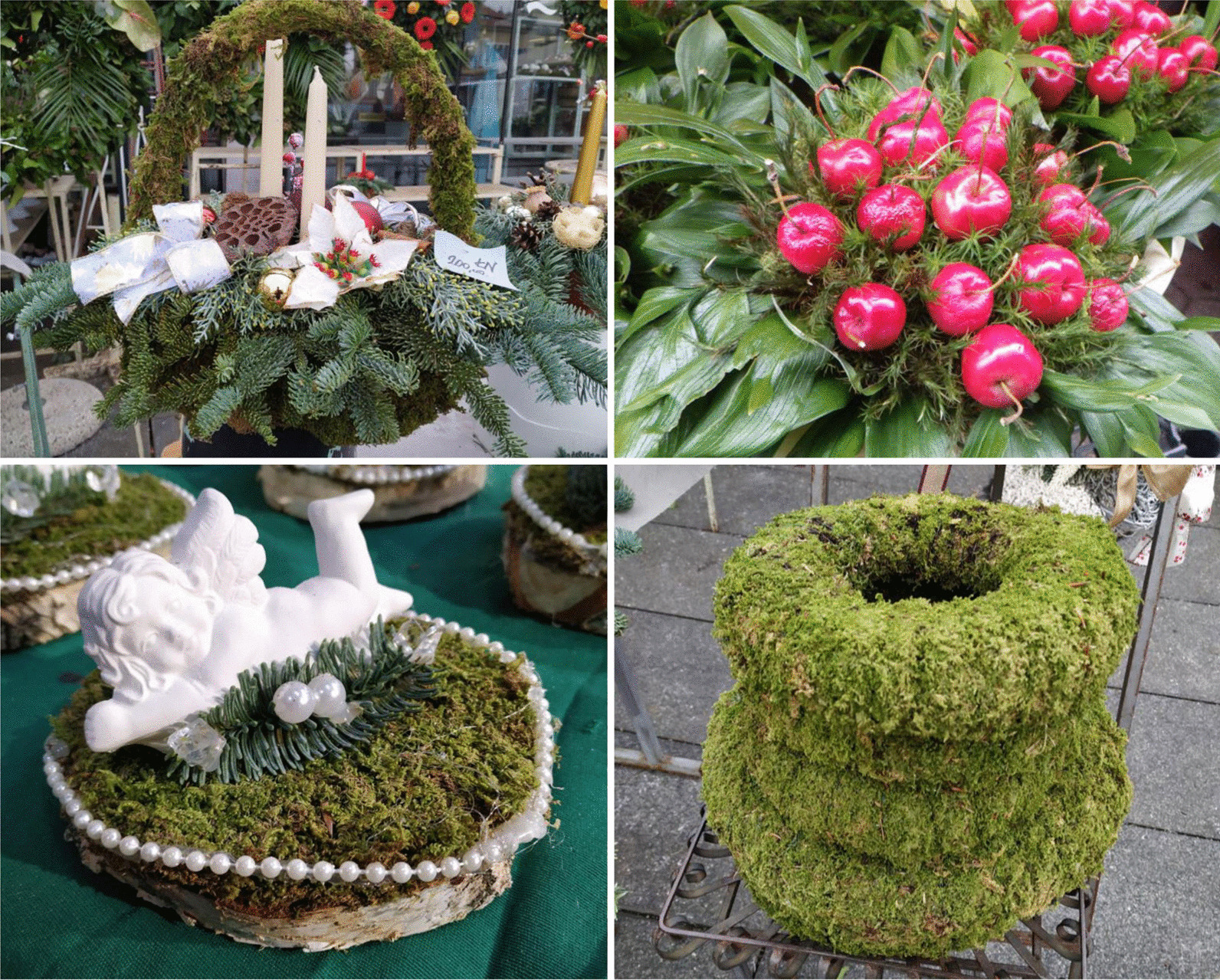
Variety of ready-made Christmas decorations using bryophytes (moss baskets, bouquets, floral arrangements with small sculptures, Christmas wreaths). Photographs by Marija Bučar

Besides the dominant carpets and cushions, bryophytes were also sold along with various vascular plants in ready-made Christmas decorations: moss baskets, Christmas wreaths, Advent wreaths (crowns), miscellaneous floral arrangements with candles, small sculptures, Christmas bulbs, etc. (Fig. 4). A similar variety of decorative usage of bryophytes during the Christmas season (with some peculiarities such as moss table runners and moss animal figures) was previously documented in the USA [55].

A total of 275 bryophyte samples (218 in Zagreb and 57 in Split) were collected on 28 farmers’ markets. A sample (collected specimens hereafter) represents a single species collected at one of the vendors. The bryophytes were sold by 78 vendors (49 in Zagreb and 29 in Split). The maximum number of vendors per market in Zagreb was registered at Dolac Market (nine vendors), while in Split the corresponding number was at the Stari Pazar market (22 vendors). Identification of the specimens resulted in a list of 47 bryophyte species. The mean number of species offered per vendor was 3.5 (min. 1, max. 12) in the entire sample. In Zagreb, the mean number of species per vendor was 4.5 (min. 1, max. 12), while in Split the mean number was 1.9 (min. 1, max. 9). The mean number of species per market was 8.0 (min. 1, max. 24) in the entire sample. In Zagreb, the mean number of species per market was 9.3 (min. 1, max. 24), while in Split the mean number was 4.8 (min. 2, max. 16).

The mosses (division Bryophyta) were represented by 43 species, belonging to 37 genera and 19 families, while only four liverwort species (division Marchantiophyta) were recorded. These were exclusively foliose species (*Frullania dilatata*, *Plagiochila porelloides*, *Porella platyphylla* and *Scapania umbrosa*) belonging to four genera and four families (Table 2), while thalloid liverworts were not recorded. Similarly, liverworts were rarely represented in previous studies (e.g. genera *Calypogeia*, *Diplophyllum, Frullania*, *Lejeunea, Lepidozia*, *Lophocolea, Plagiochila, Porella, Radula, Saccogyna* and *Scapania* [15, 18, 54].

**Table 2:** Checklist of bryophytes sampled in Zagreb and Split markets (n—number of specimens (number of samples of a single species collected at one of the vendors), TB species considered a target bryophyte species, LF—life-form, GT—growth type, ZG—Zagreb, ST—Split, Ma—mat, We—weft, Tu—tuft, De—dendroid, Fa—fan, Cu—cushion, Ac—aquatic colonial, A—acrocarpous, P—pleurocarpous)

Family	Species	n	TB	LF	GT	ZG	ST
**Liverworts**							
*Frullaniaceae*	*Frullania dilatata* (L.) Dumort	1		Ma		x	
*Plagiochilaceae*	*Plagiochila porelloides* (Torr. ex Nees) Lindenb	6		Tu, We		x	
*Porellaceae*	*Porella platyphylla* (L.) Pfeif	4		Fa, Ma		x	x
*Scapaniaceae*	*Scapania umbrosa* (Schrad.) Dumort	3		Ma		x	x
**Mosses**							
*Amblystegiaceae*	*Drepanocladus aduncus* (Hedw.) Warnst	1	+	We, Ac	P	x	
*Anomodontaceae*	*Anomodon viticulosus* (Hedw.) Hook. & Taylor	9	+	Ma	P	x	x
*Bartramiaceae*	*Bartramia pomiformis* Hedw	2	+	Tu	A	x	
*Brachytheciaceae*	*Brachythecium cirrosum* (Schwägr.) Schimp	1		Ma	P	x	
	*Brachythecium rutabulum* (Hedw.) Schimp	17	+	Ma, We	P	x	x
	*Brachythecium tommasinii* (Sendtn. ex Boulay) Ignatov & Huttunen	1		Ma	P	x	
	*Cirriphyllum crassinervium* (Taylor) Loeske & M.Fleisch	1		Ma	P	x	
	*Eurhynchium angustirete* (Broth.) T.J.Kop	9	+	We	P	x	
	*Eurhynchium striatum* (Hedw.) Schimp	5	+	We	P		
	*Homalothecium sericeum* (Hedw.) Schimp	26	+	Ma	P	x	x
	*Kindbergia praelonga* (Hedw.) Ochyra	1		Ma, We	P	x	
	*Oxyrrhynchium hians* (Hedw.) Loeske	4		Ma, We	P	x	
	*Plasteurhynchium striatulum (*Spruce) M.Fleisch	1		Ma, De	P	x	
	*Pseudoscleropodium purum* (Hedw.) M.Fleisch	4	+	We	P	x	x
	*Rhynchostegium confertum* (Dicks.) Schimp	2	Ma	Ma	P	x	
	*Rhynchostegium megapolitanum* (Blandow ex F.Weber & D.Mohr) Schimp	2	+	Ma, We	P	x	
*Bryaceae*	*Ptychostomum capillare* (Hedw.) Holyoak & N.Pedersen	2		Tu, Cu	A	x	
	*Rhodobryum ontariense* (Kindb.) Kindb	3		Tu	A	x	x
*Dicranaceae*	*Dicranum scoparium* Hedw	4	+	Tu	A	x	x
	*Dicranum tauricum* Sapjegin	1		Tu	A	x	
*Entodontaceae*	*Entodon concinnus* (De Not.) Paris	1		We	P		x
*Funariaceae*	*Funaria hygrometrica* Hedw	1		Tu	A	x	
*Hylocomiaceae*	*Loeskeobryum brevirostre* (Brid.) M.Fleisch	1		We	P	x	
	*Pleurozium schreberi* (Willd. ex Brid.) Mitt	1		We	P	x	
*Hypnaceae*	*Hypnum cupressiforme* Hedw	32	+	Ma	P	x	x
*Lembophyllaceae*	*Isothecium alopecuroides* (Lam. ex Dubois) Isov	16	+	De	P	x	x
*Leucodontaceae*	*Nogopterium gracile* (Hedw.) Crosby & W.R.Buck	1		Ma, De	P		x
*Mniaceae*	*Plagiomnium affine* (Blandow ex Funck) T.J.Kop	2		Ma	A	x	
	*Plagiomnium cuspidatum* (Hedw.) T.J.Kop	4		Ma	A	x	
	*Plagiomnium rostratum* (Schrad.) T.J.Kop	2		Ma	A	x	
	*Plagiomnium undulatum* (Hedw.) T.J.Kop	8	+	Tu, De	A	x	
*Myuriaceae*	*Ctenidium molluscum* (Hedw.) Mitt	19	+	Ma, We	P	x	x
*Neckeraceae*	*Alleniella complanata* (Hedw.) S.Olsson, Enroth & D.Quandt	8	+	Fa, Ma	P		
	*Exsertotheca crispa* (Hedw.) S.Olsson, Enroth & D.Quandt	4	+	We, Fa	P	x	x
	*Homalia trichomanoides* (Hedw.) Brid	7	+	Fa	P	x	
	*Pseudanomodon attenuatus* (Hedw.) Ignatov & Fedosov	12	+	Ma	P	x	
*Polytrichaceae*	*Atrichum undulatum* (Hedw.) P.Beauv	4	+	Tu	A	x	
	*Polytrichum formosum* Hedw	16	+	Tu	A	x	
*Pottiaceae*	*Tortella squarrosa* (Brid.) Limpr	1		Tu	A		x
	*Tortella tortuosa* (Hedw.) Limpr	2		Tu, Cu	A		x
*Pylaisiaceae*	*Calliergonella cuspidata* (Hedw.) Loeske	2		We	P	x	
*Thuidiaceae*	*Thuidium delicatulum* (Hedw.) Schimp	14	+	We, Ma	P	x	x
	*Thuidium tamariscinum* (Hedw.) Schimp	7	+	We, Ma	P	x	x

Although the tradition of using bryophytes in Christmas nativity scenes is widespread and found in many countries, there are almost no comprehensive studies of bryophyte diversity engaged in this tradition. Except for several papers referring to Central and North America [17, 54, 56] and focusing at best on a few dominant taxa, the only extensive survey of bryophyte diversity was performed in northern Spain, in which 66 taxa (54 mosses and 12 liverworts) were recorded [18], 23 of them in common with our research. However, instead of collecting data on farmer’s markets, the Spanish study focused on 26 already made nativity sets found in churches, private businesses and private residences.

**Fig. 5:**
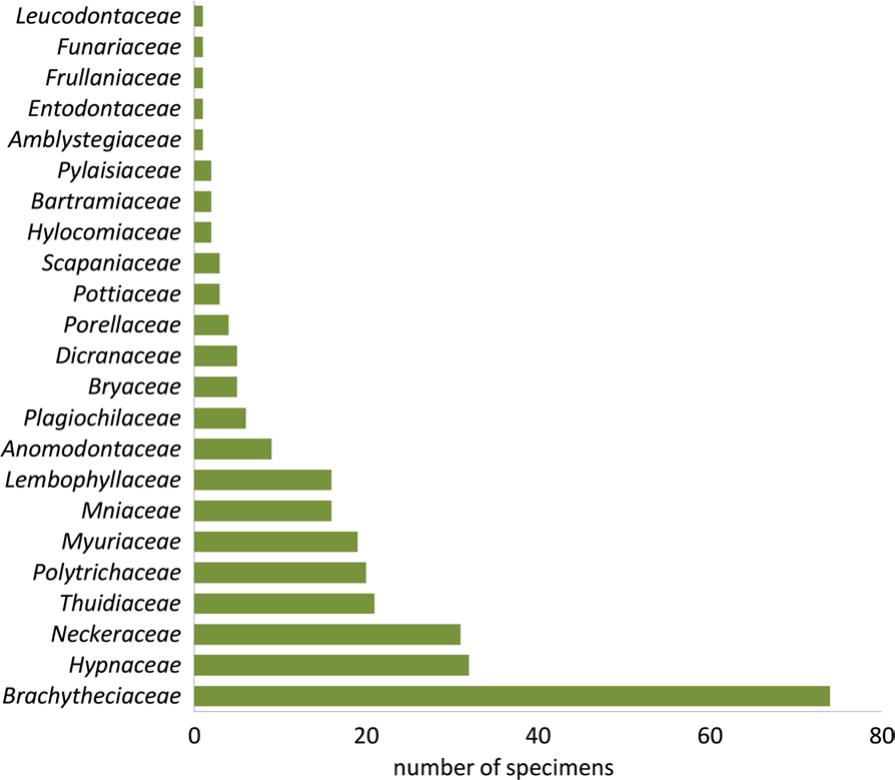
The number of collected specimens of bryophyte families

In terms of the number of species, *Brachytheciaceae* was the most represented out of the 23 recorded families in our research. It was represented by 13 species, followed by *Mniaceae* and *Neckeraceae* with four species each (Fig. 5). Other families were represented with either one or two species only. Similarly, the best-represented family in the Spanish study was also *Brachytheciaceae*, with 13 species [18]. In terms of the number of collected specimens, *Hypnaceae,* although represented by only one species (*Hypnum cupressiforme*), was the second most harvested family after *Brachytheciaceae* (Fig. 1). Similarly, *Thuidiaceae*, *Polytrichaceae* and *Myuriaceae* were represented with only one or two species but were more frequently harvested.

**Fig. 6:**
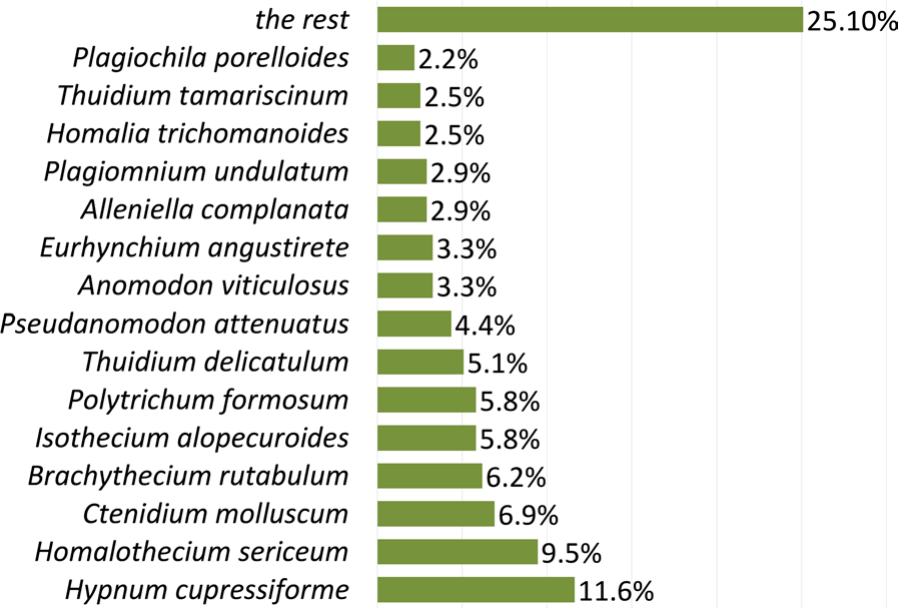
Harvesting frequency of bryophyte species

The most frequently harvested species in our study were *Hypnum cupressiforme* (11.6%), *Homalothecium sericeum* (9.5%) and *Ctenidium molluscum* (6.9%) (Fig. 6). By contrast, in the Spanish study, the most frequent species, accounting for as much as 65% of the samples, were *Thuidium tamariscinum*, *Pseudoscleropodium purum*, *Hypnum cupressiforme* and *Eurynchium striatum* (all found on Croatian markets as well) [18]. The genus *Hypnum*, along with *Thuidium*, seems to be the most frequently chosen taxon in Christmas decorations in the Americas [15, 17, 53, 54, 56]. However, in all cases, the preferred moss species were selected because of their rather large size and pleurocarpous growth type, i.e. forming bryophyte carpets, as well as because of their overall attractiveness, which makes them preferable for nativity scene decoration. The dominant species in assemblages of the bryophytes present in individual moss carpets or cushions sold in Croatian markets were the most probably selected by the vendors as target species (Table 2), opposed to accidentally collected species that were intertwined in rather small amounts among the stems and branches of the target species. There was a relatively high share of target species (46.8%, 22 species) recognized in this study and these were exclusively moss species, i.e. pleurocarpous mosses.

In our study, approximately two-thirds (67.4%) of recorded species are pleurocarpous, while their harvesting frequency (a share of particular species specimens in a total number of collected specimens) is as high as 80.1%. Accordingly, 32.6% of recorded species are acrocarpous, with a harvesting frequency of 19.9%. Very similar ratios were reported from Spain—63% of pleurocarpous and 37% of acrocarpous mosses [18]. Pleurocarpous mosses which branch out extensively such as *Hypnum cupressiforme*, *Homalothecium sericeum*, *Ctenidium molluscum*, *Brachythecium rutabulum*, etc. are seemingly favoured for Croatian Christmas decorations (Fig. 6). They form sheet-like mats that stick together upon harvesting [55], whereas acrocarpous mosses form cushions, tufts or grow as scattered individuals [45]. Furthermore, epiphytic species such as *Alleniella complanata* and *Homalia trichomanoides* (Fig. 8), which grow in fans, i.e. form carpets in one plane on a vertical substrate such as tree bark, are considered suitable as well. The only other somewhat more frequent acrocarpous species is *Polytrichum formosum* (harvesting frequency 5.8%), which is selected for its size and resemblance to small trees (Fig. 8). Some other rather distinctive acrocarpous mosses such as *Plagiomnium* species were sampled accidentally along with pleurocarps as *Hypnum* or *Ctenidium* by being intertwined with their mat-like forms. *Alleniella complanata* and *Polytrichum formosum* were also among the frequently selected species in Spain [18].

**Fig. 7:**
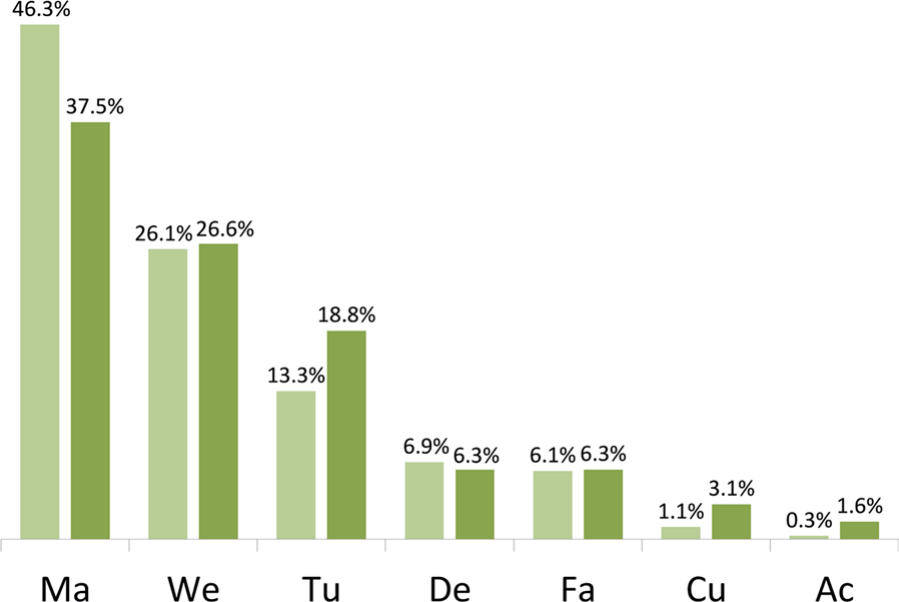
Harvesting frequency of different life-forms (bright green) and life-form frequency within species pool (dark green) (Ma—mat, We—weft, Tu—tuft, De—dendroid, Fa—fan, Cu—cushion, Ac—aquatic colonial)

Considering the life forms, the most represented species are those growing in cohesive forms such as mats, wefts and tufts (Fig. 7). Over 64% of recorded species grow as mats and wefts, while the overall harvesting frequency of these forms was as high as 72%. The most popular life forms in the Mexican sample were wefts and cushions [15] and while wefts are well represented in our research, cushions only account for 1.1% of the total sample.

Vendors in Split reported collecting bryophytes in the Mediterranean part of Croatia, within Split and its hinterland, Dalmatinska Zagora (e.g. Mt. Mosor and the surroundings of the town of Sinj) (average 20 km distance), while vendors in Zagreb collected their material in a wider area of the inland part of Croatia (regions of Zagorje, Prigorje, Lika, and Turopolje) (average 32 km distance). In the Mexican study of Oaxaca Christmas plant market, the vendors reported collecting usually nearby their residences, within an hour and a half from their villages [17] while in Spain most nativity set creators affirmed that their harvest took place along the paths surrounding their homes and villages [18]. Most of the Croatian vendors said they did not distinguish among moss species, except for the obvious difference between carpets and cushions. Thus, no vernacular names for bryophytes were documented in this study.

**Fig. 8:**
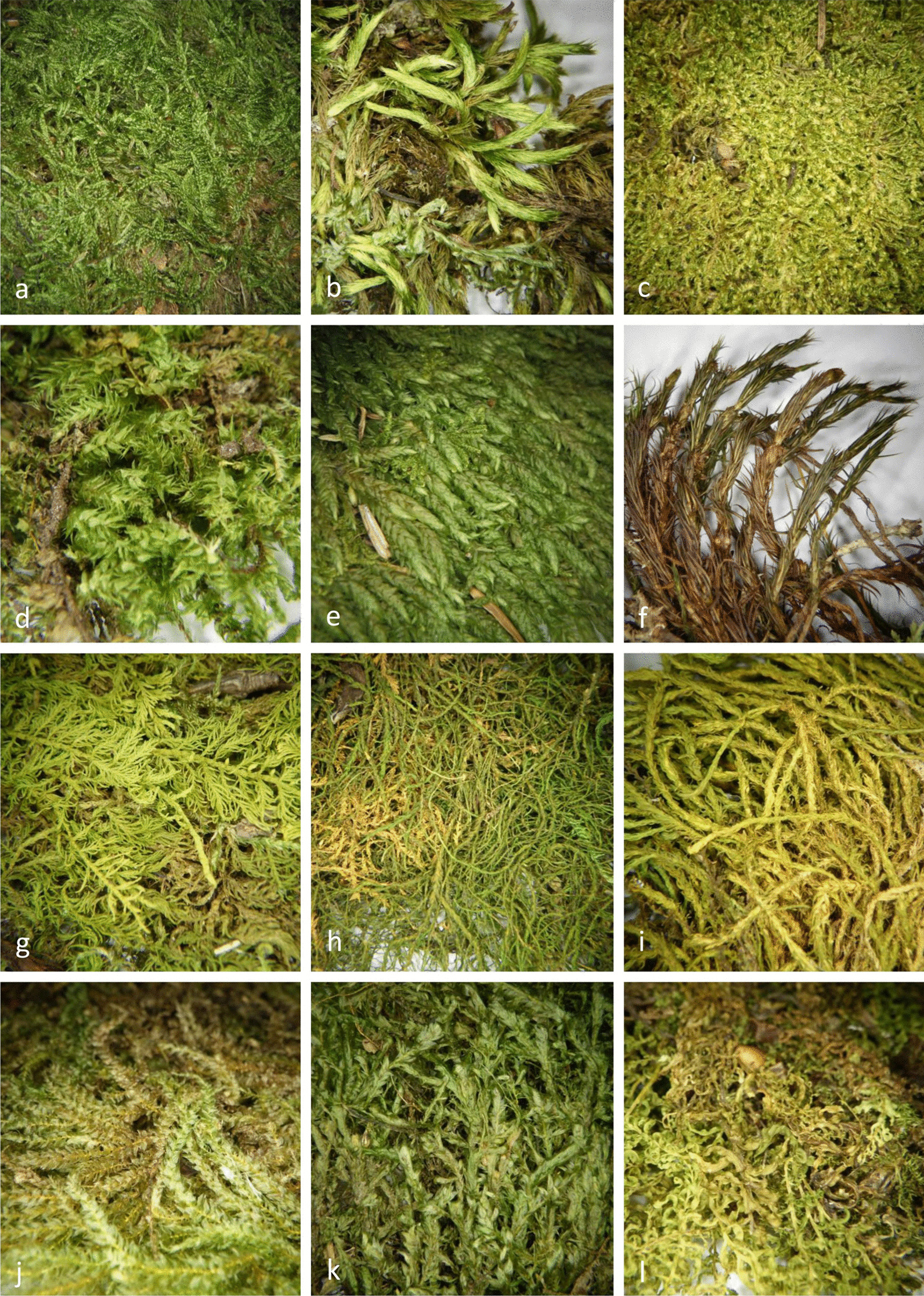
The most commonly harvested bryophytes (**a**—*Hypnum cupressiforme*, **b**—*Homalothecium sericeum*, **c**—*Ctenidium molluscum*, **d**—*Brachythecium rutabulum*, **e**—*Isothecium alopecuroides*, **f**—*Polytrichum formosum*, **g**—*Thuidium delicatulum*, **h**—*Pseudanomodon attenuatus*, **i**—*Anomodon viticulosus*, **j**—*Eurhynchium angustirete*, **k**—*Alleniella complanata*, **l**—*Plagiomnium undulatum*). Photographs by Marija Bučar

The overall offer of bryophytes and decorations made of bryophytes was more diverse and abundant in Zagreb, where 49 vendors on 15 farmers’ markets sold goods containing 43 species. However, in Split, at six farmers’ markets and from informal standalone market stalls on the streets only 29 vendors sold goods, containing 18 species. In a comparison of the most common species in the inland and Mediterranean samples, the Zagreb samples were more diverse. About half of all Zagreb samples (53.2%) consists of eight species (*Hypnum cupressiforme*, *Brachythecium rutabulum*, *Polytrichum formosum*, *Isothecium alopecuroides*, *Ctenidium molluscum*, *Thuidium delicatulum*, *Pseudanomodon attenuatus* and *Eurhynchium angustirete*), while approximately half of Split samples (54.4%) comprises only two species (*Homalothecium sericeum* and *Hypnum cupressiforme*) (Fig. 8). The two cities have only 14 species in common, whereas four were only found in Split (*Entodon concinnus*, *Nogopterium gracile*, *Tortella squarrosa* and *T. tortuosa*) and 29 exclusively in Zagreb (Table 2).

Vendors in Zagreb claimed that they harvested from more diverse habitats (i.e. forests, along the streams) and substrates (forest ground, rocks, tree bark, tree trunks, tree base and dead wood), while collectors in Split mostly referred to harvesting in forests and from rocks. In other studies, some ecological groups of bryophytes, such as epiphytes and epilithic mosses, have been generally recognized as preferred in commercial moss harvesting. In the western part of the USA (state of Oregon), epiphytic bryophytes are nowadays increasingly being removed to supply a global multimillion-dollar floral industry. These activities may pose a serious threat to the particular bryophyte species, as well as forest ecosystems in general imposing a need for research into the ecosystem roles of epiphytic bryophytes [57]. Epiphytic moss mats with a surface area of at least 100 cm^3^ are considered harvestable by commercial harvesters in Northwestern Oregon where those mats are usually composed of species such as *Isothecium myosuroides*, *Neckera douglasii*, *Eurhynchium oreganum* etc., and liverworts such as *Frullania* and *Porella* [14]. Although their congenial pairs were found in our research, they were not frequent in our samples. Interestingly, in the eastern part of the USA (West Virginia) [13], it was found that the majority (60%) of bryophytes were harvested from rocks, with the species *Hypnum imponens, H. curvifolium* and *Thuidium delicatulum* usually being targeted.

A significant number of species (all liverworts, most acrocarpous mosses and some pleurocarpous), which are less attractive to harvesters, was collected non-intentionally, entangled in carpets of other, more frequent species. The same was observed in Spain, where nearly 44% of species from nativity sets were assigned as accidental, randomly collected [18]. The more striking case was reported from the eastern part of the USA (state of West Virginia), although not within the Christmas harvest. In that study, as many as 73 incidentally harvested bryophytes were identified among commercially harvested *Thuidium* and *Hypnum* species [13].

In our research, two rare, accidentally harvested acrocarpous mosses were collected. The first one, *Rhodobryum ontariense*, is extremely rare in Croatia, with a single locality (island of Lošinj in the Northern Adriatic) dating back to 1999 [58]. This study revealed two new localities; one in the vicinity of the town of Ogulin in the continental part of Croatia (specimen collected at a farmers’ market in Zagreb) and the other near the town of Sinj in the Mediterranean region (specimen collected at a farmers’ market in Split). The species was collected from rocks at both sites. Unfortunately, more precise information on the localities was not given by the interviewed vendors. The second noteworthy species is *Loeskeobryum brevirostre*, known in Croatia from only several localities, but all reported more than 70 years ago. Unfortunately, we were not able to gather any data on the collection site from the vendor in Zagreb, thus we can only confirm the recent presence of species in Croatia.

While in some parts of the world extensive moss harvesting is recognized as an activity that poses a threat to natural habitats and their ecology and as such requires management practice [13–15], in Croatia, there is still no appropriate assessment of moss harvesting and its potential negative impact on the species and habitats. Our research was primarily focused on the diversity of the species harvested and sold during the Christmas holidays on traditional farmers’ markets. However, another study, still unpublished, of native vascular plant species sold on farmers’ markets throughout the year revealed that bryophytes are almost totally absent in the market offer outside the Christmas period or can be found rarely and in small quantities in floral arrangements. Compared to the intensive commercial moss harvesting in the USA as part of a large industrial market for mosses, we found the low-intensive exploitation of bryophytes from natural populations in Croatia acceptable in terms of nature protection. Moreover, regarding the threat status, all 47 recorded species are considered to be of least concern (LC) in the European Red List of Mosses, Liverworts and Hornworts [51].

## Conclusions

To our knowledge, the present study gives the first perspective on the use of bryophytes in traditional nativity scenes in Croatia and Southeastern Europe, contributing to the scarce ethnobotanical documentation of the decorative and ceremonial use of bryophytes in Christmas festivities in Europe and globally.

The overall diversity of the bryophytes harvested and sold in the two largest Croatian cities is quite high with 47 species recorded. However, around 53% of the species are collected infrequently, i.e. accidentally and in very small amounts, being intertwined in the branches of more popularly harvested pleurocarpous bryophytes that form extensive mats on soil, rocks or tree basis. Considering the offer of bryophytes in Zagreb and Split, the intensity of bryophyte exploitation from natural populations in Croatia is still acceptable in terms of nature protection and no threatened species were found during this study. On the other hand, we found two very rare species—new localities for *Rhodobryum ontariense* and confirmation of *Loeskeobryum brevirostre* in Croatia after more than 70 years, which gave our study the added value.

Further research should address the use of bryophytes and vascular plants in other Christian traditions, ceremonies and rituals in Southeastern Europe. The heritage of miscellaneous traditional knowledge concerning plants in this part of Europe is in dire need of further research, which will ensure it from falling into oblivion.

## Data Availability

Voucher specimens were deposited in the bryophyte collection of Herbarium Croaticum (ZA). The original data sheet with interviews is available from the corresponding author on reasonable request.
